# Immunopathogenesis of Human T-Cell Leukemia Virus Type-1-Associated Myelopathy/Tropical Spastic Paraparesis: Recent Perspectives

**DOI:** 10.1155/2012/259045

**Published:** 2012-02-06

**Authors:** Mineki Saito, Charles R. M. Bangham

**Affiliations:** ^1^Department of Immunology, Graduate School of Medicine, University of the Ryukyus, 207 Uehara, Okinawa 903-0215, Japan; ^2^Department of Immunology, Wright-Fleming Institute, Imperial College London, Norfolk Place, London W2 1PG, UK

## Abstract

Human T-cell leukemia virus type-1 (HTLV-1) is a replication-competent human retrovirus associated with two distinct types of disease only in a minority of infected individuals: the malignancy known as adult T-cell leukemia (ATL) and a chronic inflammatory central nervous system disease HTLV-1-associated myelopathy/tropical spastic paraparesis (HAM/TSP). HAM/TSP is a chronic progressive myelopathy characterized by spastic paraparesis, sphincter dysfunction, and mild sensory disturbance in the lower extremities. Although the factors that cause these different manifestations of HTLV-1 infection are not fully understood, accumulating evidence from host population genetics, viral genetics, DNA expression microarrays, and assays of lymphocyte function suggests that complex virus-host interactions and the host immune response play an important role in the pathogenesis of HAM/TSP. Especially, the efficiency of an individual's cytotoxic T-cell (CTL) response to HTLV-1 limits the HTLV-1 proviral load and the risk of HAM/TSP. This paper focuses on the recent advances in HAM/TSP research with the aim to identify the precise mechanisms of disease, in order to develop effective treatment and prevention.

## 1. Introduction

Human T-cell leukemia virus type-1 (HTLV-1) is a human retrovirus etiologically associated with adult T-cell leukemia (ATL) [1–3] and HTLV-1-associated myelopathy/tropical spastic paraparesis (HAM/TSP) [4, 5]. HAM/TSP is a chronic progressive myelopathy characterized by spastic paraparesis, sphincter dysfunction, and mild sensory disturbance in the lower extremities [6]. Cases of HAM/TSP have been reported throughout the HTLV-1 endemic areas such as Southern Japan, the Caribbean, Central and South America, the Middle East, Melanesia, and equatorial regions of Africa [7]. Sporadic cases have also been described in nonendemic areas such as the United States and Europe, mainly in immigrants from an HTLV-1 endemic area. In contrast to HIV-1 infection, few with HTLV-1 develop disease: approximately 2%-3% of infected persons develop ATL [8] and other 0.25%–3.8% develop HAM/TSP [9–12], while the majority of infected individuals remain lifelong asymptomatic carriers (ACs). However, the ability to evaluate the individual risk of HTLV-1-associated diseases in each AC would make a significant clinical impact, especially in HTLV-1 endemic areas. During the last three decades since the discovery of HTLV-1 as the first pathogenic human retrovirus, advances in HTLV-1 research have helped us to understand the clinical features of HTLV-1 associated diseases, the virological properties of HTLV-1, and the importance of the viral, host, and environmental risk factors as well as the host immune response against HTLV-1 infection. However, the precise mechanism of disease pathophysiology is still incompletely understood, and the treatment is still unsatisfactory, because good small-animal models for studying HTLV-1 infection and its associated diseases were unavailable until recently. In this paper, we summarize the recent developments of HTLV-1 research to try to identify more precisely the pathogenetic mechanisms of the disease in order to develop effective treatment and prevention. 

## 2. HTLV-1 Infection and Clinical Features of HAM/TSP

### 2.1. Virological Aspects of HTLV-1

HTLV-1 is classified as a complex retrovirus in the genus *Deltaretrovirus* of the subfamily *Orthoretrovirinae* and infects 10–20 million people worldwide [13–15]. HTLV-1 can be transmitted through sexual contact [16], injection drug use [15], and breastfeeding from mother to child [17, 18]. For over two decades, the investigation of HTLV-1-mediated pathogenesis has been focused on Tax, an HTLV-1 encoded viral oncoprotein, since Tax has been viewed as critical for leukemogenesis because of its pleiotropic effects on both viral and many cellular genes responsible for cell proliferation, genetic instability, dysregulation of the cell cycle, and apoptosis [19]. However, Tax expression is not detected in about 60% of freshly isolated samples from ATL cases [20]. In 2002, another regulatory protein encoded in the minus or antisense strand of the virus genome, named HTLV-1 basic leucine zipper factor (HBZ), was identified [21]. The spliced form of HBZ is expressed in all ATL [22] and HAM/TSP [23] cases, and its expression is strongly correlated with the HTLV-1 proviral load (PVL) in HTLV-1-infected individuals and with disease severity in HAM/TSP patients [23]. Also, HBZ protein promotes proliferation of ATL cells and induces T-cell lymphomas in CD4^+^ T cells by transgenic expression, indicating the possible involvement of HBZ expression in the development of ATL [22, 24]. Moreover, among the HTLV-1 encoded viral genes, only the HBZ gene sequence remains intact, unaffected by nonsense mutations and deletion [25]. These findings indicate that HBZ expression is indispensable for proliferation and survival of ATL cells and HTLV-1 infected cells, and that Tax expression is not always necessary for the maintenance of ATL [26].

### 2.2. Clinical and Pathological Features of HAM/TSP

HAM/TSP is a chronic progressive myelopathy characterized by spastic paraparesis, sphincter dysfunction, and mild sensory disturbance in the lower extremities [6]. In addition to neurological symptoms, some HAM/TSP cases also exhibit autoimmune-like disorders, such as uveitis, arthritis, T-lymphocyte alveolitis, polymyositis, and Sjögren syndrome [14]. Among ACs, the lifetime risk of developing HAM/TSP, which is different among different ethnic groups, ranges between 0.25% and 4%. It has been reported that the annual incidence of HAM/TSP is higher among Jamaican subjects than among Japanese subjects (20 versus 3 cases/100,000 population), with a two to three times higher risk for women in both populations [9–12]. The period from initial HTLV-1 infection to the onset of HAM/TSP is assumed to range from months to decades, a shorter time than for ATL onset [11, 31]. HAM/TSP occurs both in vertically infected individuals and in those who become infected later in life (i.e., through sexual contact [almost exclusively from male to female], intravenous drug use, contaminated blood transfusions, etc.). The mean age at onset is 43.8 years, and the frequency of HAM/TSP is higher in women than in men (the male to female ratio of occurrence is 1 : 2.3) [11]. 

Pathological analysis of HAM/TSP autopsy materials indicates that the disease affects the spinal cord, predominantly at the thoracic level [27, 32, 33]. Loss of myelin and axons in the lateral, anterior, and posterior columns is associated with perivascular and parenchymal lymphocytic infiltration with the presence of foamy macrophages, proliferation of astrocytes, and fibrillary gliosis. In the cases with active-chronic lesions in the spinal cord, perivascular inflammatory infiltration with similar composition of cell subsets was also seen in the brain [28]. The peripheral nerve pathology of HAM/TSP patients with sensory disturbance showed varying degrees of demyelination, remyelination, axonal degeneration, regeneration, and perineurial fibrosis [29, 30]. The presence of atypical lymphocytes (so-called “flower cells”) in peripheral blood and cerebrospinal fluid (CSF), a moderate pleocytosis, and raised protein content in CSF are typically found in HAM/TSP patients. Oligoclonal immunoglobulin bands in the CSF, raised concentrations of inflammatory markers such as neopterin, tumor necrosis factor (TNF)-*α*, interleukin (IL)-6 and interferon (IFN)-*γ*, and an increased intrathecal antibody (Ab) synthesis specific for HTLV-1 antigens have also been described [34]. Clinical progression of HAM/TSP is associated with an increase in the proviral load in individual patients, and a high ratio of proviral loads in CSF cells/peripheral blood mononuclear cells (PBMCs) is also significantly associated with clinically progressive disease [35]. The clinical and pathological characteristics of HAM/TSP described above are shown in Table 1.

## 3. Risk Factors for HAM/TSP 

### 3.1. Host Genetic

A previous population association study of 202 cases of HAM/TSP and 243 ACs in Kagoshima prefecture, HTLV-1 endemic Southern Japan, revealed that one of the major risk factors is the HTLV-1 PVL. The median PVL was more than ten times higher in HAM/TSP patients than in ACs, and a high PVL was also associated with an increased risk of progression to disease [36, 37]. A higher PVL in HAM/TSP patients than in ACs was observed in other endemic areas such as the Caribbean [38], South America [39], and the Middle East [40]. It was suggested that genetic factors such as the human leukocyte antigen (HLA) genotype are related to the high PVL in HAM/TSP patients and genetic relatives. In Southern Japan, possession of the HLA-class I genes HLA-A*02 and Cw*08 was associated with a statistically significant reduction in both HTLV-1 PVL and the risk of HAM/TSP, whereas possession of HLA-class I HLA-B*5401 and class II HLA-DRB1*0101 predisposes to HAM/TSP in the same population (Table 2) [37, 41]. Since the function of class I HLA proteins is to present antigenic peptides to CTL, these results imply that individuals with HLA-A*02 or HLA-Cw*08 mount a particularly efficient CTL response against HTLV-1, which may therefore be an important determinant of HTLV-1 PVL and the risk of HAM/TSP. In fact, it has been reported that CTL spontaneously kills autologous HTLV-1-infected cells *ex vivo* [42], granzymes and perforin are more highly expressed in individuals with a low PVL [43], and the lytic efficiency of the CD8^+^ T cell response, that is, the fraction of autologous HTLV-1-expressing cells eliminated per CD8^+^ T cell per day, was inversely correlated with both PVL and the rate of spontaneous proviral expression [44]. These findings indicate that the CTL against HTLV-1 reduces PVL and risk of HAM/TSP. Moreover, using a combination of computational and experimental approaches, MacNamara et al. recently reported that a CTL response against HBZ restricted by protective HLA alleles such as HLA-A*02 or Cw*08, but not a response to the immunodominant protein Tax, determines the outcome of HTLV-1 infection [45]. 

Analysis of non-HLA host genetic factors by candidate gene approaches revealed that non-HLA gene polymorphisms also affect the risk of developing HAM/TSP (Table 2). For example, the TNF-*α* promoter-863 A allele [47] and the longer CA repeat alleles of matrix metalloproteinase (MMP)-9 promoter [48] predisposed to HAM/TSP, whereas IL-10-592 A [49], stromal-derived factor (SDF)-1 +801A, and IL-15 +191 C alleles [47] conferred protection against HAM/TSP. The polymorphisms in the MMP-9 and IL-10 promoters were each associated with differences in the HTLV-1 Tax-mediated transcriptional activity of the respective gene [48, 49]. However, the contributions of these non-HLA genes to the pathogenesis of HAM/TSP are largely unknown, and these data have not yet been reproduced in different populations. Further candidate gene studies together with genome-wide association studies in different ethnic populations in larger sample size may provide evidence for the association of non-HLA genes with HAM/TSP pathogenesis.

### 3.2. HTLV-1 Genotype and Genomic Integration Site

Although most studies of HTLV-1 genotype have reported no association between variants of HTLV-1 and the risk of HAM/TSP, Furukawa et al. reported the association between HTLV-1 *tax* gene variation and the risk of HAM/TSP [46]. The *tax* subgroup A, which belongs to cosmopolitan subtype A, was more frequently observed in HAM/TSP patients, and this association was independent of the protective effect of the HLA allele HLA-A*02. HLA-A*02 appeared to give protection against only one of the two prevalent sequence variants of HTLV-1, *tax* subgroup B which belongs to cosmopolitan subtype B, but not against *tax* subgroup A in the Japanese population [46]. Interestingly, HLA-A*02 appeared not to give protection against infection with cosmopolitan subtype A in a population in Iran [40]. Moreover, the Iranian HTLV-1 strain has a Rex protein that is 20 amino acids longer than that of the Japanese strain that belongs to cosmopolitan subtype B. Experiments are now underway to compare the functions of these Rex proteins. 

Recently, to test whether the genomic integration site determines the abundance and the pathogenic potential of an HTLV-1-positive T-cell clone, Gillet et al. reported the results of high-throughput mapping and quantification of HTLV-1 proviral integration in the host genome [50]. They mapped >91,000 unique insertion sites (UISs) of the provirus from 61 HTLV-1-infected individuals in primary PBMCs and showed that a typical HTLV-1-infected host carries between 500 and 5000 UISs in 10 *μ*g of PBMC genomic DNA. They calculated an oligoclonality index (OCI) to quantify the clonality of HTLV-1-infected cells *in vivo* and found that the OCI did not distinguish between ACs and patients with HAM/TSP and that there was no correlation between OCI and HTLV-1PVL in either ACs or HAM/TSP patients. These results indicate that the higher PVL observed in patients with HAM/TSP was attributable to a larger number of UISs but not, as previously thought, from a difference in clonality. They also obtained evidence that the abundance of established HTLV-1 clones is determined by genomic features of the host DNA flanking the provirus. Namely, HTLV-1 clonal expansion *in vivo* is favored by a proviral integration site near a region of host chromatin undergoing active transcription, or same-sense transcriptional orientation of the provirus. Negative selection of infected clones, probably by CTLs during chronic infection, favors establishment of proviruses integrated in transcriptionally silenced DNA, and this selection is more efficient in ACs than in HAM/TSP, indicating the selection of HTLV-1-infected T-cell clones with low pathogenic potential.

## 4. Immune Response to HTLV-1

### 4.1. Innate Immune Response

#### 4.1.1. Natural Killer (NK) Cells 

Previous reports indicated that patients with HAM/TSP had both a lower frequency and a lower activity of NK cells (especially the CD3^+^CD16^+^ subset) than ACs although the results were not normalized with respect to PVL [51]. Since an important mechanism of induction of NK cell-mediated killing is recognition by the NK cell of a complex of the nonpolymorphic MHC molecule HLA-E bound to a peptide derived from the signal sequence of some other MHC class I molecules, a synthetic tetramer of HLA-E with the HLA-G signal sequence peptide was used to identify NK cells in HAM/TSP patients [52]. The results showed a significantly lower frequency of HLA-E tetramer-binding cells in HAM/TSP patients than ACs, and as in the earlier studies [51], this reduction in frequency was particularly notable in the CD3^+^ cells, whereas there was no significant difference in the frequency of HLA-E tetramer-binding CD3^−^ cells between patients with HAM/TSP and ACs [52]. Recent data also suggest that the frequency of invariant NKT (iNKT) cells in the peripheral blood of HAM/TSP patients is significantly decreased when compared with healthy subjects and/or ACs [53, 54]. These findings indicate that the activity of the NK or NKT cell response was associated with the absence of HAM/TSP. Interestingly, a previous uncontrolled preliminary trial of treatment of HAM/TSP with fermented milk containing viable *Lactobacillus casei *strain Shirota resulted in a significant increase in NK cell activity, with improvements in clinical symptoms [55]. Thus, circulating NK and NKT cells might also play an important role in the disease progression and the pathogenesis of HAM/TSP. Recently, it has been reported that in addition to the previously described CD8^+^ T-cell spontaneous proliferation [56], CD56^+^ NK cells also spontaneously proliferated *in vitro*, and spontaneous NK cell proliferation positively correlated with HTLV-1 PVL but not with the presence of HAM/TSP [57]. A hallmark of HTLV-1 infection is the *in vitro* proliferation of PBMCs when cultured in the absence of exogenous antigen or mitogen, referred to as spontaneous lymphocyte proliferation (SLP), and in HAM/TSP patients, the levels of SLP reflect the severity of the disease [58, 59]. Most of the high SLP observed in PBMCs from HAM/TSP patients is likely to be explained by a greater spontaneous expression of the provirus and consequently a greater proliferation of responding CD8^+^ T cells in culture [56]. The greater proviral expression may be partly attributable to the impaired function and decreased number of NK cells in HAM/TSP patients. Although further studies are required to clarify the role of NK cells in HTLV-1 infection and HAM/TSP pathogenesis, NK cells might be also an interesting candidate for future immunotherapy.

#### 4.1.2. Interferons

Type I interferon (IFN) is a key innate immune cytokine produced by cells in response to viral infection. The type I IFN response protects cells against invading viruses by inducing the expression of interferon-stimulated genes (ISGs), which execute the antiviral effects of IFN [60]. The ISGs then generate soluble factors including cytokines that activate adaptive immunity or directly inhibit the virus itself [61]. To date, IFN-*α* is not only one of the effective therapeutic agents for HAM/TSP, but also known as an only therapeutic agent whose efficacy was demonstrated in randomized placebo-controlled trials [62, 63]. However, the therapeutic benefit is small, and IFN-*α* is not in general use in the treatment of HAM/TSP. The combination of the antiretroviral agent zidovudine (AZT) and IFN-*α* is also beneficial for overall survival in smoldering and chronic (i.e. indolent) ATL [64] although its efficacy has not yet been confirmed in well-designed prospective studies. It might be interesting to analyse which ISGs are changed in the course of IFN-*α* treatment and the functional role of ISGs as potential targets for therapy. In PBMCs of HTLV-1-infected individuals, the level of HTLV-1 mRNA is very low, and viral protein is not detectable, but these molecules are rapidly expressed after a short time in culture *in vitro * [42]. However, the mechanisms of this phenomenon are largely unknown. Recently, it has been reported that HTLV-1 expression in HTLV-1-infected T-cells is suppressed by stromal cells, that is epithelial cells and fibroblasts, in culture through type I IFNs [65]. Namely, HTLV-1 Gag protein expression was suppressed when contacted with stromal cells and restored when separated from the stromal cells. Although neutralizing antibodies against human IFN-*α*/*β* receptor only partly abrogated this phenomenon, the results indicate that the innate immune system suppresses HTLV-1 expression *in vitro* and *in vivo*, at least through type I IFN.

### 4.2. Antibody Response to HTLV-1

In 2002, it was reported that antibodies that recognize HTLV-1 Tax protein can cross-react with a heterogenous-nuclear-riboprotein (hnRNP-) A1, suggesting intriguing evidence for antigen mimicry in HTLV-1 infection [66]. However, subsequent analysis using Japanese samples under fully masked conditions indicated that there was no difference in the incidence of anti-hnRNP A1 Abs between HAM/TSP and other neurological diseases [67]. It is unlikely that anti-Tax Ab explains the onset or initial tissue damage of HAM/TSP, as the host protein hnRNP-A1 is not confined to the central nervous system but is widely expressed [68] and is not normally accessible to Ab attack. Anti-Tax Ab might be associated with subsequent inflammation following initial tissue damage and disruption of blood brain barrier, which is probably caused by the antiviral immune responses to HTLV-1 and induces the release of autoantigens. 

In HTLV-1 infection, HAM/TSP patients generally have a higher anti-HTLV-1 Ab titer than ACs with a similar HTLV-1 proviral load [69–71]. These anti-HTLV-1 Abs often include IgM in both ACs and patients with HAM/TSP [70, 71]. These findings suggest that there was persistent expression of HTLV-1 proteins *in vivo* and the existence of an augmented humoral immune response to HTLV-1 in HAM/TSP patients. Although Ab responses to the immunodominant epitopes of the HTLV-1 envelope (Env) proteins were similar in all of three clinical groups (HAM/TSP, ATL, and ACs), reactivity to four Tax immunodominant epitopes was higher in HAM/TSP patients (71%–93%) than in ATL patients (4%–31%) or ACs (27%–37%) [72]. Among these anti-HTLV-1 antibodies, anti-EnvAb is particularly important since some anti-Env Abs have neutralizing activity against HTLV-1. Antisera raised against recombinant HTLV-1 Env polypeptides [73, 74], vaccinia virus containing HTLV-1 env gene [75, 76], immunization with neutralizing epitope peptides [77], and passive transfer of human IgG that has neutralizing activity [78, 79] were all shown to neutralize HTLV-1 infectivity. In HTLV-1 infection, the roles of HTLV-1 neutralizing Ab *in vivo* are still largely unknown. It will be interesting to examine whether HTLV-1 neutralizing Ab titres correlate with disease status and PVL in infected individuals. Since the mutation rate of HTLV-1 provirus is significantly lower than HIV-1, passive immunization with human monoclonal Ab may be beneficial and effective method to prevent HTLV-1 infection.

### 4.3. Cytotoxic T-Lymphocyte (CTL) Response to HTLV-1

Previous reports indicated that the HTLV-1-specific CD8^+^ CTLs are typically abundant, chronically activated, and mainly targeted to the viral trans activator protein Tax [80]. Also, as already mentioned, the median PVL in PBMCs of HAM/TSP patients was more than ten times higher than that in ACs, and a high PVL was also associated with an increased risk of progression to disease [36, 37]. Furthermore, HLA-A*02 and HLA-Cw*08 genes were independently and significantly associated with a lower PVL and a lower risk of HAM/TSP [37, 41], and CD8^+^ T cells efficiently kill autologous Tax-expressing lymphocytes in fresh PBMCs in HTLV-1-infected individuals [42]. These data have raised the hypothesis that the class I-restricted CD8^+^ CTL response plays a critical part in limiting HTLV-1 replication *in vivo* and that genetically determined differences in the efficiency of the CTL response to HTLV-1 account for the risk for developing HAM/TSP. Indeed, as mentioned above (Section 3.1), MacNamara et al. [45] have shown that HLA class 1 alleles which strongly bind oligopeptides from the HBZ protein enable the host to make a more effective immune response against HTLV-1; therefore, such individuals have a lower PVL and are more likely to be asymptomatic. Moreover, another recent report showed the presence of HBZ-specific CD4^+^ and CD8^+^ cells *in vivo *in patients with HAM/TSP and in ACs and a significant association between the HBZ-specific CD8^+^ cell response and asymptomatic HTLV-1 infection [81]. These findings provide strong evidence to support the hypothesis of the crucial role of CTLs and also confirm the importance of HBZ for persistent infection.

Since the frequency of HTLV-1-specific CD8^+^ T cells was significantly higher in HAM/TSP patients than ACs [82, 83], and these cells have the potential to produce proinflammatory cytokines [84], there is a debate on the role of HTLV-1-specific-CD8^+^ T cells, that is, whether these cells contribute to the inflammatory and demyelinating processes of HAM/TSP, or whether the dominant effect of such cells *in vivo* is protective against disease. The analysis of gene expression profiles using microarrays in circulating CD4^+^ and CD8^+^ lymphocytes indicated that granzymes and perforin are more highly expressed in individuals with a low PVL [43], suggesting that a strong CTL response is associated with a low PVL and a low risk of HAM/TSP. Indeed, the lytic capacity of HTLV-1-specific CTL in patients with HAM/TSP and ACs, quantified by a CD107a mobilization assay, showed significantly lower CD107a staining in HTLV-1-specific CTL in HAM/TSP than ACs [85]. Recently, it has been reported that the high CTL avidity, which is closely associated with the lytic efficiency of CTL, correlates with low PVL and proviral gene expression [44], indicating that the efficient control of HTLV-1 *in vivo* depends on the quality of CTL, which determines the position of virus-host equilibrium and also the outcome of persistent HTLV-1 infection. However, two caveats must be made here. First, a protective role and a pathogenic role of CTLs are not mutually exclusive. Indeed, there are other examples of viral infections in which the virus-specific CTLs exert both beneficial (antiviral) and detrimental (inflammatory) effects, such as lymphocytic choriomeningitis virus (LCMV) infection in the mouse [86]. Second, it is difficult to separate cause and effect in analyzing the association between T-cell attributes and the efficiency of viral control in a persistent infection at equilibrium.

### 4.4. CD4^+^ Helper T-Cell Response to HTLV-1

Antiviral CD4^+^ T-cell responses are of central importance in driving B-cell and CD8^+^ T-cell responses *in vivo*. The most common HTLV-1 antigen recognized by CD4^+^ T-cells is the Env protein [87, 88], in contrast with the immunodominance of Tax in the CD8^+^ T-cell response [89–91]. At a similar PVL, patients with HAM/TSP had significantly increased frequency of virus-specific CD4^+^ T cells compared to ACs [88, 92]. The antiviral T-helper (Th)1 phenotype is also dominant among HTLV-1-specific CD4^+^ T cells in both ACs and patients with HAM/TSP [93], and there is a higher frequency of IFN-*γ*, TNF-*α*, and IL-2 production by CD4^+^ T cells in patients with HAM/TSP compared to AC of a similar PVL [93, 94]. A role for CD4^+^ T cells in initiating and causing HAM/TSP is also consistent with the immunogenetic observations that the possession of HLA-DRB1*0101, which restricts the immunodominant epitope of HTLV-1 Env gp21, was associated with susceptibility to HAM/TSP in independent HTLV-1-infected populations in Southern Japan [37, 41] and Northeastern Iran [40]. Accordingly, a synthetic tetramer of DRB1*0101 and the immunodominant HTLV-1 Env380-394 peptide was used to analyze Env-specific CD4^+^ T cells directly *ex vivo* [92]. The results showed that the frequency of tetramer^+^CD4^+^ T cells was significantly higher in HAM/TSP patients than ACs with similar PVL. Furthermore, direct ex vivo analysis of tetramer^+^CD4^+^ T cells from two unrelated DRB1*0101-positive HAM/TSP patients indicated that certain T-cell receptors (TCRs) V*β*s were utilized and antigen-specific amino acid motifs were identified in complementarity determining region (CDR) 3 from both patients. These results suggest that the observed increase in virus-specific CD4^+^ T cells in HAM/TSP patients, which may contribute to CD4^+^ T cell-mediated antiviral immune responses and to an increased risk of HAM/TSP, was not simply due to the rapidly growing HTLV-1-infected CD4^+^ T cells but was the result of *in vivo* selection by specific MHC-peptide complexes, as observed in freshly isolated HLA-A*0201/Tax11-19 tetramer^+^CD8^+^ T cells [95] and muscle-infiltrating cells from HAM/TSP patients and HTLV-1-infected polymyositis patients [96].

### 4.5. Regulatory T Cells (Tregs) in HTLV-1 Infection

Regulatory T cells (Tregs) are important mediators of peripheral immune tolerance and also play an important role in chronic viral infections. In HTLV-1 infection, it has been reported that HTLV-1 preferentially and persistently infects CD4^+^CD25^+^ lymphocytes *in vivo* [97], which contain the majority of the Foxp3^+^ Tregs [98]. In HAM/TSP patients, the frequency of Foxp3^+^ expression in CD4^+^CD25^+^ cells is lower than that in ACs and uninfected healthy controls [97, 99]. This is probably due to the fact that CD25 is transcriptionally induced by HTLV-1 Tax [100], which may result in the reduced proportion of Foxp3^+^ cells in the CD4^+^CD25^+^ population in HTLV-1-infected individuals, especially HAM/TSP patients. It is important to note that the CD4^+^CD25^+^ population contains a mixture of Tregs and activated non-Tregs. Therefore, it is inappropriate to use CD25 as a marker of Tregs in HTLV-1 infection: the best current working definition of Treg phenotype is CD4^+^Foxp3^+^. Reports from different geographic regions indicate that the percentage of CD4^+^Foxp3^+^ cells is higher in the HAM/TSP patients than in ACs [101–103]. It has been reported that the high frequency of CD4^+^Foxp3^+^T cells in HTLV-1-infected individuals is maintained by CCL22 produced by HTLV-1-infected PBMCs [104]. The frequency of HTLV-1-negative CD4^+^Foxp3^+^ cells was positively correlated with the HTLV-1 proviral load [102, 105], and the CTL activity was negatively correlated with the frequency of HTLV-1-negative CD4^+^Foxp3^+^ cells [102], suggesting that CD4^+^Foxp3^+^ Tregs may impair the CTL surveillance of HTLV-1. If this is the case, activity of CD4^+^Foxp3^+^ cells may also determine the risk of developing HAM/TSP via increasing the HTLV-1 PVL.

### 4.6. Dendritic Cells (DCs)

Dendritic cells are antigen-presenting cells which play a critical role in the regulation of the adaptive immune response. In HTLV-1 infection, it has been shown that the DCs from HAM/TSP patients were infected with HTLV-1 [106], and the development of HAM/TSP is associated with rapid maturation of DCs [107]. As already mentioned, one of the hallmarks of HTLV-1 infection is the spontaneous lymphocyte proliferation (SLP). Interestingly, depletion of DCs from the HAM/TSP patient's PBMCs abolished SLP, whereas supplementing DCs restores proliferation [106]; supplementing B cells or macrophages had no effect. A DC-dependent mechanism of SLP was further supported by data showing that antibodies to MHC class II, CD86, and CD58 can block SLP [108]. Recently, it has been demonstrated that both myeloid and plasmacytoid DCs are susceptible to infection with cell-free HTLV-1, and HTLV-1-infected DCs can rapidly transfer virus to autologous primary CD4^+^ T cells [109]. In addition, other groups have obtained evidence that HTLV-1 transmission from DCs to T cells was mediated primarily by DC-SIGN [110], and DCs play a major part in generating and maintaining the Tax-specific CD8^+^ T cells both *in vitro* and *in vivo* [111]. Moreover, using transgenic mouse models that permit conditional transient depletion of CD11c^+^ DCs, and a chimeric HTLV-1 that carries the envelope gene from Moloney murine leukemia virus, Rahman et al. demonstrated the critical role of DCs in their ability to mount both innate and adaptive immune responses during early cell-free HTLV-1 infection [112, 113]. Since HTLV-1 can impair the differentiation of monocytes into DCs [114], the interaction of DCs with HTLV-1 plays a central part in the persistence and pathogenesis of HTLV-1.

## 5. Concluding Remarks

**Figure 1:**
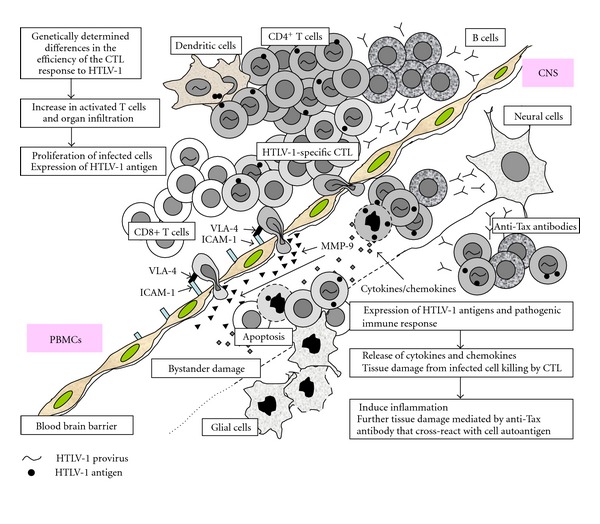
Hypothesis for the pathogenesis of human T-cell leukemia virus type-1 (HTLV-1) -associated myelopathy/tropical spastic paraparesis (HAM/TSP). Accumulating evidence suggests that the virus-host immunologic interactions play a pivotal role in HAM/TSP pathogenesis. Genetically determined less efficient CTL response against HTLV-1 may cause higher proviral load and antigen expression in infected individuals, which lead to activation and expansion of antigen-specific T-cell responses, subsequent induction of large amounts of proinflammatory cytokines and chemokines, and progression of HAM/TSP development. It is also possible that the immunoglobulin G specific to HTLV-1-Tax, which cross-react with heterogeneous nuclear ribonuclear protein-A1 (hnRNP-A1), is associated with subsequent inflammation following initial tissue damage.

As shown in Figure 1, accumulating evidence suggests that the host immune response, especially the CTL response, plays a critical role in determining the risk of HAM/TSP. A less efficient CTL response against HTLV-1 may cause a higher PVL and higher antigen expression in infected individuals, which in turn lead to activation and expansion of antigen-specific T-cell responses, subsequent induction of large amounts of proinflammatory cytokines and chemokines, and progression to HAM/TSP. Since HLA class 1 genotype determines only up to 50% of HAM/TSP risk in infected people [41], it is important to discover other factors that determine the efficiency of the CTL response to HTLV-1 and the outcome of HTLV-1 infection. Studies of the HTLV-1 receptor and DCs are also critical in the development of vaccine approaches to elicit cellular immune responses to key viral proteins such as Tax and Env to ablate HTLV-1-infected T cells. Newer approaches using genetically engineered and/or humanized mouse models for HTLV-1 infection will help to develop effective treatment and prevention of HAM/TSP in the future.

## Figures and Tables

**Table 1:** Clinical and pathological characteristics of HAM/TSP.

Clinical characteristics		References
Onset	Insidious, slowly progressive	[11]

Major clinical symptoms	Spastic paraparesis	[11]
	Sphincter dysfunction	
	Mild sensory disturbance in the lower extremities	

Complications	Uveitis	[14]
	Arthritis	
	T-lymphocyte alveolitis	
	Polymyositis	
	Sjögren syndrome	

Mean age at onset	43.8 years	[11]

Male-to-female ratio	1 : 2.3 (male : female)	[11]

Laboratory data	Positive anti-HTLV-1 antibody in both serum and CSF	[11]
	Moderate pleocytosis and raised protein content in CSF	

Pathological characteristics		References

Spinal cord	Loss of myelin and axons in the lateral, anterior, and posterior columns-predominantly at the thoracic level	[27]
	Perivascular and parenchymal lymphocytic infiltration with the presence of foamy macrophages, proliferation of astrocytes, and fibrillary gliosis-predominantly at the thoracic level	

Brain	Perivascular and parenchymal lymphocytic infiltration with the presence of foamy macrophages, proliferation of astrocytes, and fibrillary gliosis	[28]
	Perivascular inflammatory infiltration and fibrosis only in the cases with active-chronic lesions in the spinal cord. The composition of cell subsets was similar both in the spinal cord and in the brain	

Peripheral nerve	Varying degrees of demyelination, remyelination, axonal degeneration, regeneration, and perineurial fibrosis	[29, 30]

**Table 2:** Host genetic and viral factors associated with the risk of HAM/TSP.

Factor	Condition	Effect	Reference(s)
Viral factors	HTLV-1 *tax* subgroup A	Susceptible	[46]
	Proviral load	Susceptible	[36]

*Host factors*			
HLA	A*02	Protective	[37, 41]
	Cw*08	Protective	[41]
	B*5401	Susceptible	[41]
	DRB1*0101	Susceptible	[37]

Non-HLA	TNF-*α* promoter −863 A allele	Susceptible	[47]
	longer CA repeat alleles of MMP-9 promoter	Susceptible	[48]
	IL-10 promoter −592 A allele	Protective	[49]
	SDF-1 promoter +801 A allele	Protective	[47]
	IL-15 +191 C allele	Protective	[47]
